# Editorial

**DOI:** 10.1080/17571472.2016.1216728

**Published:** 2016-08-16

**Authors:** Jean Ledger

**Affiliations:** ^a^ King’s College London School of Management and Business

London Journal of Primary Care brings together clinical and non-clinical knowledge to help understand issues that sit at the interface between clinical practice, public health and policy. As such, the journal remains open to multidisciplinary sources of knowledge and opinion whilst offering a focal point for a dispersed learning community to share findings from research. This approach is evidenced in this Issue.

Morton and colleagues span public health, sports sciences and primary care; they come together to benchmark knowledge levels of physical activity recommendations among patient groups. Lawless et al. observe the difficulties in evaluating service interventions in primary care across multiple sites, bringing together unique perspectives from commissioning, public health and general practice. Latif and colleagues describe how a voluntary organization supported health education in the British-Bangladeshi community. We also welcome Professor Francesco Carelli who reviews the life of artist, Sonia Delaunay.

LJPC will continue to welcome papers such as these that illuminate the rich multidisciplinary nature of primary care.

Dr Jean Ledger *King’s College London School of Management and Business* Jean.Ledger@kcl.ac.uk

